# State and trait serotonin variations interact to shape the intrinsic connectivity and gradient architecture of the brain – a combined TPH2 genetics and tryptophan depletion study in men

**DOI:** 10.1007/s00213-026-07029-4

**Published:** 2026-02-24

**Authors:** Lan Wang, Congcong Liu, Xianyang Gan, Keith Kendrick, Weihua Zhao, Christian Montag, Ting Xu, Benjamin Becker

**Affiliations:** 1 The Center of Psychosomatic Medicine, Sichuan Provincial People’s Hospital, Sichuan Provincial Center for Mental Health, University of Electronic Science and Technology of China, Chengdu, China; 2 School of Life Science and Technology, University of Electronic Science and Technology, Chengdu, China; 3 School of Psychology, Xinxiang Medical University, Xinxiang, China; 4 Centre for Cognitive and Brain Sciences, Institute of Collaborative Innovation, and, Department of Psychology, University of Macau, Macau , Macau SAR; 5 Faculty of Psychology, Southwest University, Chongqing, China; 6 Key Laboratory of Cognition and Personality, Ministry of Education, Chongqing, China; 7 MIND & AI Lab, Department of Psychology; Strategic Research Theme (SRT): AI, Society & Social Dynamics, The University of Hong Kong, Hong Kong , Hong Kong S.A.R., China

**Keywords:** Serotonin, Tryptophan f, Tryptophan hydroxylase, VmPFC, Amygdala

## Abstract

**Rationale:**

Serotonin (5-HT) critically regulates cognitive and emotional functions, and both stable and transient variations in 5-HT signaling have been associated with emotional dysregulations. However, findings regarding the neurofunctional effects of transient 5-HT variations have been highly inconsistent.

**Objective:**

Therefore, we examined whether individual variations in a central 5-HT-regulating genetic polymorphism (tryptophan hydroxylase 2, TPH2) represent a vulnerability or resilience factor for the effects of acute tryptophan depletion (ATD) on functional brain architecture.

**Methods:**

The current study utilized a pharmacogenetic within-subject randomized placebo-controlled resting-state fMRI design with *N* = 53 healthy male participants in combination with spontaneous intrinsic neural activity, functional connectivity, and connectome gradient analyses to compare the neurofunctional effects of ATD-induced transient reduction in central 5-HT signaling between TPH2 genotypes (a priori genotyping for rs4570625, GG *n* = 25 vs. TT *n* = 23).

**Results:**

ATD induced significant increases in spontaneous neural activity in hippocampal CA1 irrespective of genotype and enhanced communication of this region with the bilateral amygdala and the vmPFC specifically in GG carriers. ATD sharpened the intrinsic connectome gradient architecture in several large-scale networks, including the salience, frontoparietal, and default mode network.

**Conclusions:**

Our results identify a potential genetic marker for an increased vulnerability to the neural effects of transient variations in 5-HT signaling on the functional architecture of an anxiety- and stress-related brain circuit in men. Gradient architecture results underscore the regulatory role of 5-HT on the intricate organization of large-scale networks involved in emotional reactivity and regulation.

**Supplementary Information:**

The online version contains supplementary material available at https://doi.org/10.1007/s00213-026-07029-4.

## Introduction

Synthesized from amino acid tryptophan in the raphe nuclei (Salvan et al. 2023), serotonin (5-HT) exerts a profound influence on affective processes via its dense projections to a core limbic-prefrontal network, including the amygdala, hippocampus, and frontal cortex (Etkin et al. 2015; Liu et al. 2021a; Luo et al. 2024). Dysfunction in serotoninergic neurotransmission is an established candidate mechanism for emotional dysregulation characteristic of stress-related and anxiety disorders (Azmitia and Segal 1978; Challis and Berton 2015).

5-HT signaling is influenced by stable, trait-like, individual differences, including genetics (e.g., Booij et al. 2015) and by transient variations in the production of 5-HT in the brain (e.g., influenced by stress or nutrition, Markus 2008; van der Stelt et al. 2004). A established experimental procedure to probe the putative role of 5-HT in emotional processes is acute tryptophan depletion (ATD), a procedure leading to a transient decrease in central tryptophan and serotonergic signaling (Van Donkelaar et al. 2011). Converging evidence indicates that ATD modulates the circuits critical for emotion such that ATD-administration enhances amygdala reactivity to negative stimuli (Passamonti et al. 2012; Van der Veen et al. 2007) and alters functional connectivity within the amygdala-hippocampal-prefrontal circuits (Bär et al. 2020; Cools et al. 2005). Together these findings have provided converging evidence for 5-HT’s pivotal role in regulating limbic-prefrontal circuits that govern threat reactivity and emotion regulation (Etkin et al. 2015; Gothard 2020).

However, recent meta-analytical studies did not find strong evidence for robust effects of ATD on emotional and neural processing in healthy individuals (Raab et al. 2016; Schopman et al. 2021) suggesting that its impact on emotion and impulsive behavior is notably limited compared to the robust effects on the neurochemical level (Bîlc et al. 2023). While these findings challenge a series of observations on ATD-induced emotional and neural changes, accumulating findings from recent studies suggest considerable individual variations in the effects of ATD and that these vary as a function of trait-like and genetic factors which may mirror individual variations in the basal function and architecture of the monoamine, including the 5-HT system (e.g., Booij et al. 2015; Eisner et al. 2017; Kanen et al. 2021) and polymorphisms in the tryptophan hydroxylase 2 (TPH2) gene (Liu et al. 2023a). The TPH2 encodes the rate-limiting enzyme responsible for central serotonin synthesis from tryptophan and individual differences in the TPH2 have been associated with serotonin synthesis rates in limbic and prefrontal brain regions (Booij et al. 2012; Furmark et al. 2016), alongside variations in emotional responsiveness and regulation (Gutknecht et al. 2007; Laas et al. 2017). Specifically, the TPH2 rs4570625 single nucleotide polymorphism (SNP), situated within the gene’s promoter region (Wigner et al. 2018), with variations on this SNP being associated with individual differences in the intrinsic functional organization of the brain, including variations in amygdala functional connectivity (Liu et al. 2021b) and macroscale whole-brain structural connectivity (Markett et al. 2017). Consequently, variations in TPH2 rs4570625 SNP may underlie individual differences in the functional communication of critical emotion processing hubs in the brain and may furthermore mediate individual differences in the sensitivity of the effects of transient decrease in 5-HT signaling via ATD on these networks.

To this end the current study utilized a pre-registered within-subject randomized placebo-controlled double-blind resting state fMRI (rsfMRI) design in a sample of *N* = 53 healthy male participants to exploratorily investigate the underlying regulatory mechanisms of TPH2 rs4570625 SNP on the neurofunctional sensitivity to an ATD-induced transient reduction of central 5-HT signaling, specifically on the vmPFC-hippocampus-amygdala circuitry and whole brain gradient architecture of the brain. To determine the interactions between ATD and genotype extreme groups of the TPH2 rs4570625 SNP were sampled (TT vs. GG). We initially conducted a voxel-wise fractional amplitude of low-frequency fluctuations (fALFF) analysis - a measure closely linked to spontaneous intrinsic neural activity and with a high sensitivity to metabolic activity and pharmacological receptor engagement (e.g., Deng et al. 2022) – to determine regions sensitive to 5-HT variations with a high spatial resolution. Next, network level changes were examined using seed-based voxel-wise functional connectivity (FC) analysis to further identify interactions between genetic variation and acute depletion of 5-HT. Finally, we employed a gradient architecture analysis to determine variations in large-scale subnetwork architecture. We hypothesized that ATD-induced reduction in 5-HT signaling would (1) evoke regional-specific changes in spontaneous neural activity and connectivity in the vmPFC-hippocampus-amygdala systems; and (2) modulate the large-scale gradient architecture of the brain; and that (3) these neural effects would vary as a function of TPH2 gene polymorphism.

## Method

### Participants

53 right-handed, healthy male participants were recruited in total from the Chengdu Gene Brain Behavior Project in which saliva collection and genotyping was performed for *n* = 660 subjects (Sindermann et al. 2018). To reduce variance related to sex differences, e.g., related to sex differences in central 5-HT synthesis rates and the cognitive effects of ATD (Nishizawa et al. 1997; Sambeth et al. 2007; Ellenbogen et al. 1996), the present study recruited male individuals only. Details in the procedures are described in our previous task-based fMRI study on threat reactivity using the same protocols and sample (Liu et al. 2023a). Please note that the task-paradigm followed the resting state acquisition reported here, and two participants only underwent the resting state scan due to the decision not to participate in a threat paradigm (sample size in the present study *n* = 53, previous *n* = 51). All participants were free from a current or a history of medical, neurological or psychiatric disorder, regular use of psychotropic substances or MRI contraindications. 5 subjects were excluded due to excessive head movement (> 2 mm translation or 2° rotation, *n* = 3) and technical failure of the MRI system (*n* = 2), leading to a final sample of *n* = 48 participants for the current analyses (age range 18–27 years, mean age = 21.94 ± 2.59 years, *n* = 23 TT carriers). In line with the design of our previous study genotypic homozygotic groups were chosen, such that a comparable large number of TT vs. GG participants (23 vs. 25) were recruited from a genetic data bank. This approach helps to increase the statistical power while testing for genetic effects of rs4570625 or interaction with treatment effects, respectively. Details on the recruitment procedures and genotyping procedures are provided in our previous study (Liu et al. 2023a). The present manuscript reports the findings from the resting state fMRI assessment. The studies were approved by the local ethics committee and in accordance with the latest revision of the Declaration of Helsinki. All subjects provided written informed consent after being fully informed about the experimental procedures. Study protocols were pre-registered on clinicaltrials.gov (https://clinicaltrials.gov/ct2/show/NCT03549182, ID NCT03549182).

### Assessment of potential confounders

Given that emotional state and childhood experience can influence neural processing or central 5-HT signaling and can interact with TPH2 genetics (Ansorge et al. 2004; Duncan et al. 2015; Hahn et al. 2011; Soares et al. 2013; Liu et al. 2021b), trait and state anxiety (State-Trait Anxiety Inventory, STAI, Spielberger 1970), current stress (Perceived Stress Scale, PSS, Cohen et al. 1983) and exposure to early life stress (Childhood Trauma Questionnaire, CTQ, Bernstein et al. 2003) were assessed before treatment administration to control for differences between the genotype groups. The validated Chinese versions of these assessment were utilized in the current study (Huang et al. 2020; Shek 1988; Zhao et al. 2005). To assess effects of treatment on subjective anxiety levels the SAI was repeatedly administered before administration of the amino acid drink (T1), during transient 5-HT decrease (immediately before MRI acquisition, T2) and at the end of the experiment (T3).

### Procedure

Experimental and treatment procedures were identical to our previous study that reported the results of the task-based (threat exposure) fMRI paradigm that followed the resting state fMRI assessment. The studies aimed at determining whether TPH2 genotype can render individuals vulnerable to the neurofunctional effects of ATD during threat exposure (published as Liu et al. 2023a) or on the intrinsic brain functional architecture. To this end, extreme groups of the TPH2 rs4570625 were recruited (GG vs. TT carriers) and underwent a within-subject randomized double-blind placebo-controlled pharmaco-rsfMRI design. Eligible participants were administered an oral amino acid mixture (ATD, consisted of 5.5 g l-alanine, 4.9 g l-arginine, 2.7 g l-cystine, 3.2 g glycine, 3.2 g l-histidine, 8 g l-isoleucine, 13.5 g l-leucine, 8.9 g l-lysine, 3.0 g l-methionine, 12.2 g l-proline, 5.7 g l-phenylalanine, 6.9 g L‑serine, 6.5 g l-threonine, 6.9 g l-tyrosine, 8.9 g l-valine, total weight, 100 g), which causes a transient and selective reduction in central 5-HT levels, or a control placebo drink (identical ingredients plus 2.3 g of l-tryptophan, total weight, 102.3 g) in a randomized order during two experimental sessions. Participants were instructed to abstain from alcohol and caffeine for 24 h and from food and drinks (except water) for 12 h prior to the experiment. To adhere to the pharmacodynamic profile of treatment, participants arrived between 7:30 to 10:00 AM and underwent fMRI acquisition between 13:00 to 15:30 PM. Upon arrival, participants received a standardized protein-poor diet for breakfast. Participants next underwent a previously validated tryptophan depletion protocol with ATD which has been demonstrated to lead to a robust transient reduction in central 5-HT signaling (Moreno et al. 2010; Salomon et al. 2011; Williams et al. 1999; Yatham et al. 2012) or placebo (PLC). Previous studies have shown that administration of an ATD causes a sustained decrease in serotonin levels, with the effect reaching a maximum after approximately 5 h and lasting for 2 h (Williams et al. 1999). Therefore, participants underwent rsfMRI acquisition 5 h after treatment administration (duration 7.5 min) followed by the task-based fMRI paradigms. All subjects received both ATD and PLC treatments with an interim wash-out period of approx. 5 weeks.

### MRI acquisition and preprocessing

MRI data were acquired on a 3.0 Tesla GE MR750 system (General Electric Medical System, Milwaukee, WI, USA). To exclude subjects with apparent brain pathologies and improve normalization of the functional times series, T1-weighted high-resolution anatomical images were acquired with a spoiled gradient echo pulse sequence: repetition time (TR) = 5.9ms; flip angle = 9°; field of view (FOV) = 256 × 256 mm; acquisition matrix = 256 × 256; slice thickness = 1 mm; number of slices = 156. Resting-state data fMRI data were acquired using a T2*-weighted echo planar imaging sequence utilizing following parameters: TR = 2000ms; TE = 30ms; FOV = 220 × 220 mm; flip angle = 90°; image resolution = 64 × 64; thickness = 3.2 mm.

MRI data were preprocessed using the GRETNA package (https://www.nitrc.org/projects/gretna, Wang et al. 2015). implemented in MATLAB R2014a. A standardized preprocessing pipeline referred to GRETNA manual and was employed and included discarding the first volumes acquired during the initial 10 s for each participant, slice timing correction, realignment, spatial normalization to the Montreal Neurological Institute (MNI) space, spatial smoothing with a Gaussian Kernel of 6 mm full width at half maximum (FWHM), temporally linear detrending to diminish the effects of drift or trends in the signal, nuisance regression, temporal filtering (0.01–0.1 Hz) and scrubbing to reduce the effects of head motion.

### Region of interest (ROI) definition

Based on our regional specific hypotheses based on prior studies our analyses focused on the hippocampus, amygdala and vmPFC. In accordance with our previous study the hippocampus and amygdala ROIs were derived from Automatic Anatomical Labelling (Tzourio-Mazoyer et al. 2002). The vmPFC was defined based on an automated analysis of 199 studies using NeuroSynth (www.neurosynth.org*)* with the search term “vmPFC” and thresholded at *p* < 0.05 with false discovery rate correction (FDR, i.e. p_FDR_<0.5). Given the anatomical and functional heterogeneity of the vmPFC (Zhou and Becker 2025) we employed a functionally defined vmPFC mask (see also Liu et al. 2023a; Zhou et al. 2019 for validation of this approach). The used Neurosynth-based vmPFC mask contained a total of 1117 voxels, detailed regions and voxels information are included in Supplementary Information Table S1.

### Voxel-wise fractional amplitude of low-frequency fluctuations (fALFF) analysis

The fractional amplitude of low-frequency fluctuations (fALFF) quantifies the relative contribution of frequency fluctuations within a specific frequency band to the whole detectable frequency range (Zou et al. 2008) which is closely related to regional spontaneous neuronal activity (Chen et al. 2015). Moreover, fALFF has shown a robust replicability of pharmacological modulation of spontaneous brain activity (Xu et al. 2024). To initially examine effects of genotype and acute 5-HT reduction on spontaneous neural activity we initially calculated fALFF maps for each subject based on unfiltered preprocessed data. Next contrast maps between ATD and PLC treatment conditions were produced for each participant using z-transformed fALFF (zfALFF) maps and statistical parametric mapping software (SPM12, Wellcome Department of Imaging Neuroscience, London, UK; http://www.fil.ion.ucl.ac.uk/spm/software/spm12*).* Subsequently, we employed a voxel-wise one-sample t test to examine general effects of treatment and a two-sample t test examine interactive effects between treatment (coded in the individual contrast map as within-subject factor) and GG and TT genotype (coded as between-subject factor group) within SPM12. Results that passed *p* < 0.05 at a cluster-level family wise error (FWE) threshold in combination with an initial threshold of *p* < 0.001 uncorrected at the voxel level within our search mask were reported.

### Functional connectivity analysis

Based on the results of the fALFF analysis, we calculated a seed-based voxel-wise functional connectivity matrix performed using the User-Friendly Functional Connectivity software (UF^2^C, https://www.lniunicamp.com/uf2c*)*, which was developed by (de Campos et al. 2016) and implemented in MATLAB. Specifically, a 6 mm-radius sphere centered on the MNI peak coordinates of the identified effects on spontaneous neural activity served as a seed region and we examined positive and negative FC by extracting an average time-series from the seed and subsequently calculating the Pearson’s correlation coefficient between the time-series of the seed and each voxel in whole brain to generate correlation maps. The contrast maps and statistical analyses of z-transformed positive and negative FC compared between ATD and PLC treatment were separately performed as described above in fALFF analyses section. Similarly, results were thresholed at a cluster-level of *p* < 0.05 with FWE after small volume correction (an initial voxel-level threshold at *p* < 0.001).

### Gradient architecture analysis

To further explore the effects of variations in the 5-HT system and THP2 gene on the hierarchy of cortical organization on the whole-brain level, we constructed individual functional connectomes at the voxel level (Xia et al. 2022a). We first resampled the preprocessed brain images to 4 × 4 × 4 mm isotropic resolution, and then calculated the voxel-wise connectivity matrix between 18,933 grey matter nodes. The gradient architecture was constructed by applying diffusion map embedding approach for each subject to capture the top 10 components. Next, for each gradient map, three global metrics, including gradient explanation ratio, gradient range and gradient variation, were generated for all subjects. A general linear model with age as a covariate was employed to explore the treatment and genotype group differences. The results for the global metrics were reported at a statistical significance threshold of *p* < 0.05. In line with previous gradient studies, the results for regional gradient score maps were thresholded at a voxel level of *p* < 0.001 with Gaussian random field (GRF) correction at the cluster level of *p* < 0.05.

## Results

### Sample characteristics, confounders, and treatment effects on subjective anxiety levels

The genotype samples exhibited comparable age, stress (CTQ, PSS) and pre-treatment anxiety levels (STAI) suggesting that the TT and GG carriers in the present study were matched in terms of demographics and pre-treatment emotional state (see Table 1). Consistent with our previous findings, a trend interaction effect between genotype and treatment was observed (*p* = 0.058) with simple post-hoc tests indicating between group differences at T2 (*p* = 0.051) and T3 (*p* = 0.044). Specifically, in GG carriers, participants reported higher anxious arousal level following ATD treatment as compared to TT carriers.

**Table 1:** Sample demographics and potential confounders

	TT (*n* = 23)	GG(*n* = 25)	T, p
Age	21.87 ± 2.49	22.00 ± 2.72	-0.17,0.86
CTQ	40.00 ± 8.67	37.76 ± 4.84	1.09,0.28
PSS	13.35 ± 5.31	14.48 ± 5.61	-0.72,0.48
TAI	38.43 ± 8.41	36.72 ± 8.53	0.70,0.49
SAI_ATD_	34.00 ± 8.43	34.72 ± 9.18	-0.286,0.78
SAI_PLC_	34.83 ± 7.82	32.52 ± 7.38	1.05,0.30

Abbreviation: CTQ, Childhood Trauma Questionnaire; PSS, Perceived Stress Scale; TAI, Trait Anxiety Inventory; SAI, State Anxiety Inventory; SAI_ATD_, pre-treatment assessment during acute tryptophan depletion session; SAI_PLC_, pre-treatment assessment during placebo session

### The effects of the reduction of 5-HT signaling via ATD on spontaneous neural activity

**Fig. 1:**
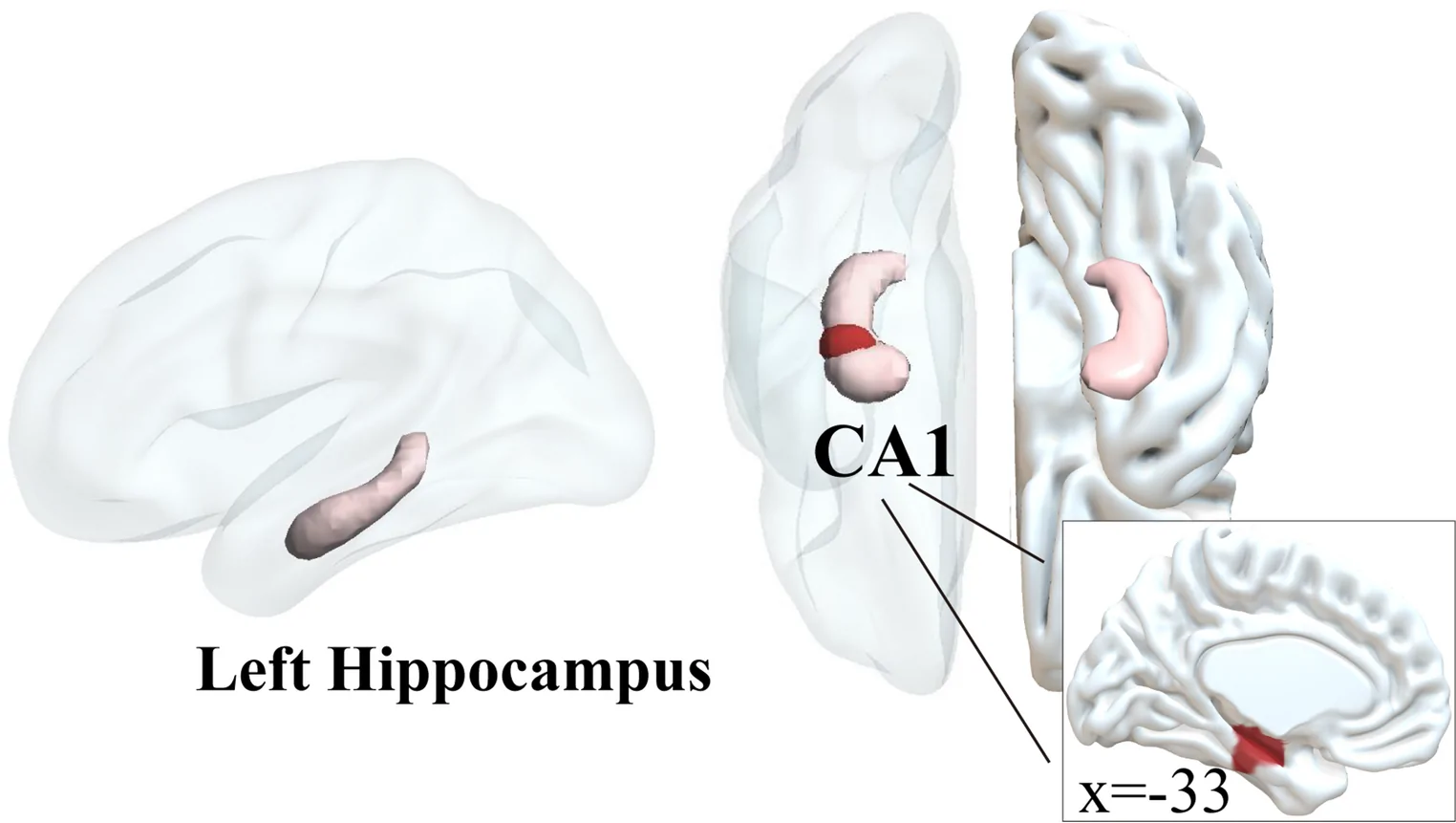
Effects of tryptophan depletion on voxel-wise spontaneous brain activity (for display purpose shown at *p* < 0.005) Left plot displays the anatomical location of the hippocampus, and the right plot presents the localization of the effects of ATD-induced increased 5-HT signaling on spontaneous activity in CA1 region of left hippocampus (T = 4.47, k = 6, p_FWE_ < 0.05 within the vmPFC-amygdala-hippocampus ROI, peak MNI, x, y, z = -33, -21, -18.)

FALFF analysis was performed to precisely map the effects of ATD on regional intrinsic neural activity. A comparison between ATD and PLC treatment revealed a significant main effect of treatment on the intrinsic activity of the left ventral hippocampus (Fig. 1), reflecting that compared to PLC transient reduction in 5-HT signaling via ATD enhanced spontaneous neural activity in this region. To facilitate a more specific localization of the effects, we utilized probabilistic maps from the Anatomy2.2b toolbox which revealed that the effect was specifically located in the CA1 region of hippocampus (Fig. 1). No interactive effects between treatment and genotype were found. The results remained consistent and significant after controlling motion parameters, CTQ, STAI and PSS, detailed results can be found in Supplementary Information Table S2.

### Functional connectivity changes for main effects of ATD and its interaction with genotype

**Fig. 2:**
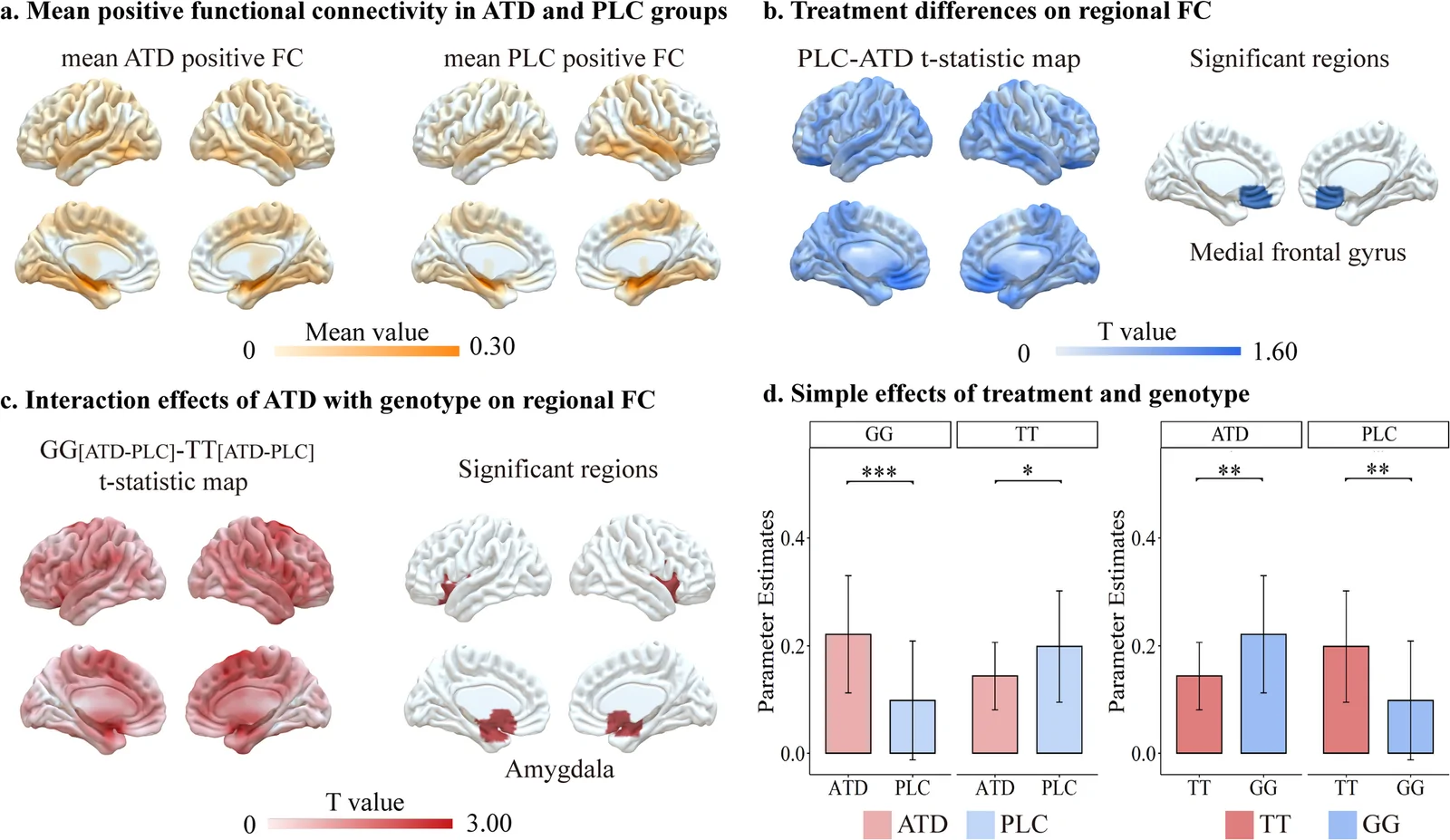
Effects of ATD treatment and its interaction effects with genotype on local functional connectivity (for display purpose shown at *p* < 0.005) **(a)** Regional mean positive FC maps in the ATD and PLC groups, respectively. **(b)** Statistical t-map between ATD and PLC group, and regional alterations in positive FC induced by treatment (T = 4.10, k = 59, p_FWE_ = 0.021, peak MNI, x, y, z = 0, 30, -15). **(c)** Changes of positive functional coupling for interaction effects of treatment with genotype (cluster 1, T = 4.19, k = 23, p_FWE_ = 0.017, peak MNI, x, y, z = 18, 0, -15; cluster 2, T = 4.09, k = 48, p_FWE_ = 0.023, peak MNI, x, y, z = -24, 3, -18). **(d)** Examination of simple effects for interaction effects with extracted parameter estimates form significant regions. * indicates *p* < 0.05, ** indicates *p* < 0.01, *** indicates *p* < 0.001

Based on the results of treatment effects on intrinsic activity, we quantified the functional connectivity matrix between the identified CA1 region (6-mm radius sphere centered at the peak coordinates x, y, z = -33, -21, -18) and the whole brain. We initially examined the functional connectivity of this region by pooling the data from the treatment sessions, reflecting that the region exhibited widespread connectivity with the bilateral fronto-parietal network, limbic network and sensorimotor network (displayed as Fig. 2a). Examining the main effects of treatment on the local network level revealed that ATD significantly decreased positive FC between the CA1 medial frontal regions, including medial frontal and orbitofrontal regions as well as further cingulate regions (Fig. 2b). A significant interaction effect between genotype and ATD was determined on the positive connectivity of the CA1 with the bilateral amygdala spreading into the parahippocampal gyrus, as well as vmPFC regions (Fig. 2c). The results remained consistent and significant after controlling motion parameters, CTQ, STAI and PSS, detailed results can be found in Supplementary Information Table S3-S4. We next extracted parameter estimates from significant regions to explore the complex interaction effect in SPSS22.0 (Fig. 2d). Bonferroni corrected post-hoc analyses on the extracted parameter estimates from significant regions revealed that in GG carriers, ATD treatment significantly enhanced functional connectivity between the CA1 with the amygdala and vmPFC (*p* < 0.001), while in the TT carriers ATD treatment decreased the connectivity between these regions (*p* = 0.047).

### ATD-induced alterations in gradient architecture on the large-scale network level

**Fig. 3:**
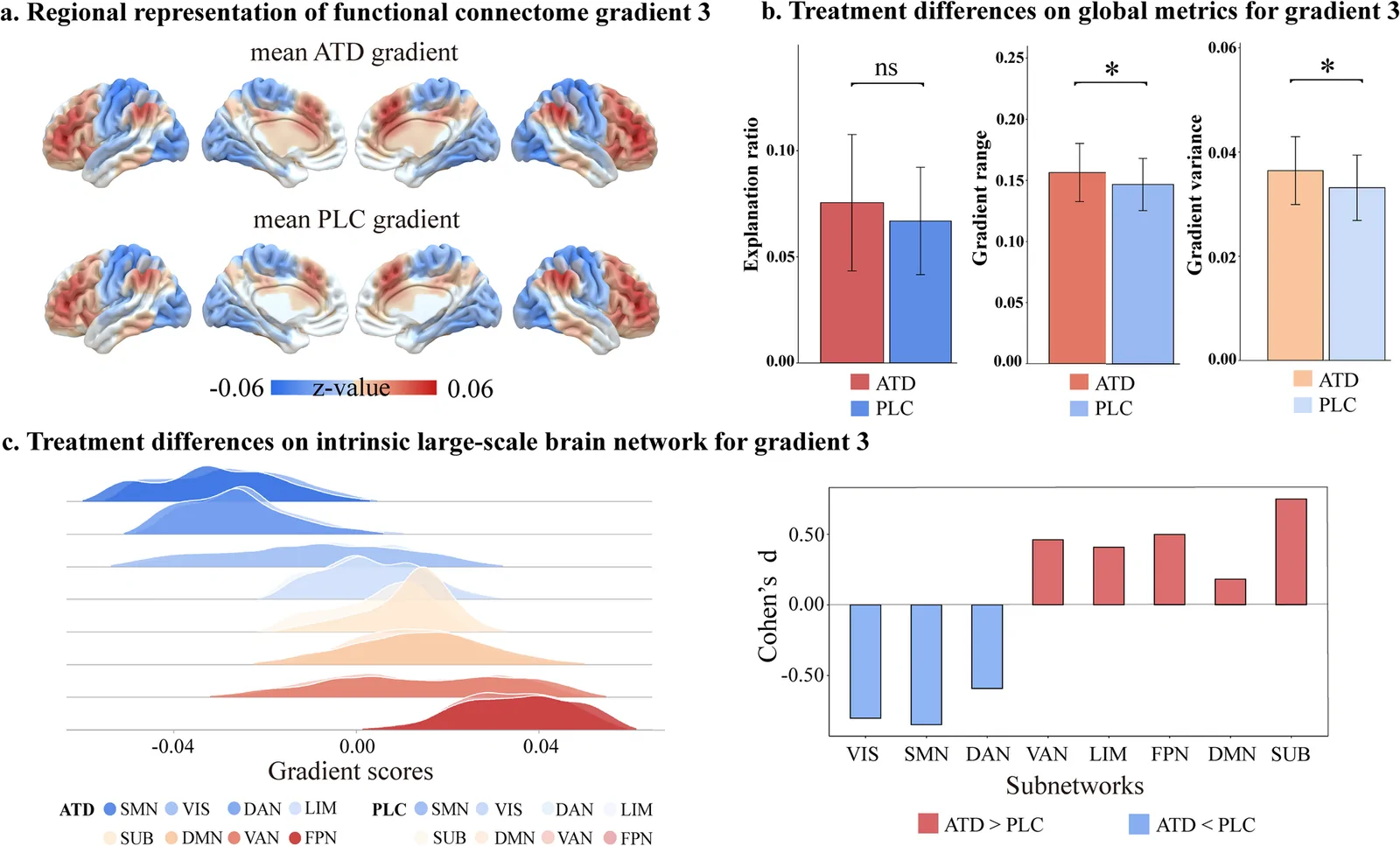
Gradient architecture mapping in ATD and PLC groups** (a)** averaged-group gradient maps for the third gradient in ATD and PLC. **(b)** Statistics for global metrics, i.e. explanation ratio, gradient range, gradient variance of gradient 3. **(c)** treatment-induced differences in intrinsic large-scale brain network for gradient3. The left ridgeline plot showed the differences of density distribution for gradient scores from eight subnetworks in ATD and PLC groups, all of which met the statistical significance level and survived the Bonferroni FDR correction (all ps < 0.0001). The right bar plot depicts the effect size cohen’s d for the difference between ATD and PLC groups in eight subnetwork systems. * indicates *p* < 0.05, ns indicates not significant. SMN, sensorimotor network; VIS, visual network; DAN, dorsal attention network; LIM, limbic network; SUB, subcortical regions; DMN, default mode network; VAN, ventral attention network; FPN, frontoparietal network

We capitalized on the diffusion map embedding method to capture gradient architecture patterns in both groups. The principal gradient 3 was represented along a gradual axis from sensorimotor network (SMN) to frontoparietal network (FPN) in both groups (Fig. 3a and c). Exploratory analyses suggest potential treatment effects on the global topology of gradient 3, with increases in both gradient range (*p* = 0.04, Cohen’s d = 0.44) and gradient variance (*p* = 0.02, Cohen’s d = 0.54, Fig. 3b). However, these global measures did not survive correction for multiple comparisons, and should therefore be interpreted with caution and considered as exploratory. In gradient 3, we found moreover a robust effect of ATD treatment on the intrinsic large-scale networks, specifically, following ATD (compared to PLC) treatment significant higher gradient scores were observed in the ventral attention network (VAN or salience network, *p* < 0.0001, Cohen’s d = 0.46), limbic network (LIM, *p* < 0.0001, Cohen’s d = 0.41), FPN (*p* < 0.0001, Cohen’s d = 0.50), default mode network (DMN, *p* < 0.0001, Cohen’s d = 0.18), subcortical network (SUB, *p* < 0.0001, Cohen’s d = 1.45), while lower gradient scores were observed in the visual network (VIS, *p* < 0.0001, Cohen’s d = -0.80), SMN (*p* < 0.0001, Cohen’s d = 0.85), dorsal attention network (DAN, *p* < 0.0001, Cohen’s d = -0.59) in ATD group (Fig. 3c). No other significant main effects of genotype (all ps > 0.076) and its interaction with treatment (all ps > 0.138) were found.

## Discussion

The present study aimed at determining whether individual variations in genetic polymorphisms directly related to individual variations in central serotonin synthesis rates (TPH2) influence the susceptibility to the effects of a transient decrease in 5-HT signaling on the intrinsic neural activity within limbic-prefrontal circuits, their connectivity and the whole brain gradient architecture of the brain. To this end we recruited extreme genotype groups of a prominent TPH2 variation that underwent a pre-registered within-subject placebo-controlled pharmacological fMRI trial. Exploratory findings revealed a trend gene-environment interaction such that GG carriers exhibited elevated state anxiety following ATD at the behavioral level. At the neural level, significant effects of ATD on spontaneous neural activity in hippocampal CA1 and functional coupling with vmPFC, hippocampus and amygdala. When accounting for the genetic differences, significant interaction effects between TPH2 genotype and ATD were observed, such that ATD specifically increased FC between hippocampus, amygdala and vmPFC in GG carriers, while it decreased FC in this pathway in the TT carriers. Finally, the current study, for the first time, revealed the effects of ATD on the brain internal gradient architecture, particularly in the third gradient, highlighting the role of 5-HT system in modulating the large-scale networks.

The effects of the reduction of 5-HT signaling via ATD on spontaneous neural activity specifically affected the hippocampal CA1 region which exhibits dense serotonergic innervation (Schneck et al. 2021) and established role in the processing of emotional stimuli (Fusar-Poli et al. 2007). This regional specificity may be explained through 5-HT1A receptor dynamics. Previous studies have elucidated that the genetic ablation of the 5-HT1A receptor precipitates alterations in synaptic activity and signal transduction, particularly within the hippocampal CA1 region promoting augmented neuronal excitability (Freeman-Daniels et al. 2011; Gordon et al. 2005). The 5-HT1A receptor serves as an inhibitory autoreceptor, rendering it susceptible to stress and responsive to antidepressants (Richardson-Jones et al. 2010) and is implicated in the modulation of hedonic processes (Hori et al. 2024). ATD, which precipitates a reduction in serotonergic signaling, attenuates the activation of 5-HT1A receptors (Cahir et al. 2007), and may thereby potentiate the spontaneous activity of hippocampal CA1 neurons. Overall, the present findings suggest robust and regional-specific effects of 5-HT modulation on spontaneous regional activity in the hippocampus, while no effects in the amygdala and vmPFC or modulatory influences of genotype were observed.

Network-level findings elucidate the interplay between state and trait serotonin variations in shaping hippocampal connectivity. ATD decreased the positive functional connectivity between the identified hippocampal region with bilateral medial frontal areas encompassing orbitofrontal and anterior cingulate regions, aligned with the established role of serotonin in modulating hippocampal-prefrontal communication, particularly during emotion regulation (Passamonti et al. 2012; Phillips et al. 2001; Unal et al. 2015). Critically, this ATD effect was modulated by TPH2 gene polymorphism, with GG carriers exhibited increased hippocampal-amygdala-vmPFC connectivity under ATD. The genotype-dependent response may partly contributed to the lack of robust effects of ATD on neural processing in recent large-scale meta-analyses (Raab et al. 2016; Schopman et al. 2021). The observed hyperconnectivity likely reflects a heighted bottom-up anxiety signaling cascade trigger by low serotonin. The hippocampus, amygdala, and prefrontal cortex constitute a tightly integrated circuit crucial for threat detection and contextual fear processing (Marcinkiewcz et al. 2016; Surget and Belzung 2022). Within this circuit, the vmPFC typically exerts top-down inhibitory control to regulate anxiety (Padilla-Coreano et al. 2019; Parfitt et al. 2017), whereas the hippocampus and amygdala convey affective information back to the prefrontal cortex, forming a feedback loop that underlies bottom-up responses to stress and anxiety (Zhang et al. 2021). This mechanism is consistent with models of anxiety disorders and PTSD (Marin et al. 2017; Morey et al. 2016; Liu et al. 2023b; Xu et al. 2021), where similar hyperconnectivity in this circuit is linked to heightened affective responses (Marcinkiewcz et al. 2016; Nawa and Ando 2019). The increased connectivity after transient dysfunction in serotoninergic system thus may signal modulation in a bottom-up anxiety signaling system. Further, this bottom-up 5-HT sensitive anxiety-related pathway was susceptible to genetic variations in the TPH2 polymorphisms. In line with our recent study (Liu et al. 2023a), GG carriers were more sensitive to the effects of transient decrease in central 5-HT signaling. The G-allele of rs4570625 SNP has been linked to reduced tryptophan hydroxylase activity (Lin et al. 2007) and impaired inhibitory control (Booij et al. 2012), potentially amplifying serotonin depletion effects. The genotype-specific effects may indicate that genetic predispositions significantly influence how serotonin depletion impacts neural connectivity, possibly representing a compensatory mechanism to maintain emotional homeostasis in the face of diminished serotonergic tone (Reichert et al. 2019). However, as maladaptive changes in the same circuits have been reported future studies combining behavioral measures with neuroimaging data determine whether this neural characteristic is ultimately adaptive or maladaptive.

On the other hand, prior investigations have shown that stress decreases the level of 5-HT in prefrontal cortex and hippocampus (Lu et al. 2019). ATD therefore partly mimics a stress-induced attenuation of serotonergic neurotransmission, as such allowing the identification of TPH2 variants that may confer heightened vulnerability to 5-HT signaling attenuation. Within this context the present findings support a diathesis–stress model in which the TPH2 GG genotype constitutes a heritable risk factor and GG carriers exhibit pronounced alterations in hippocampus-amygdala-vmPFC communication during an ATD-induced attenuation of 5-HT signaling which translates into increased affective liability (Belsky and Pluess 2009).

Our finding that ATD specifically modulated the organization of the third functional gradient provides a novel, system-level account for the effects of ATD on the overarching functional brain architecture. This result converges with recent evidence showing a sensorimotor-to-transmodal hierarchy dysfunction in disorders linked to 5-HT dysfunction (Dong et al. 2023). Previous studies indicate that this gradient represents a hierarchical axis from sensorimotor (SMN) to transmodal (e.g., FPN) regions, and is fundamental for integrating external sensory information with internal cognitive and affective states (Marguliesa et al. 2016; Xia et al. 2022a). The ATD-induced shift in gradient 3 may represent a marker for variations in 5-HT signaling and reflect alterations in the integration between sensory information and cognitive-emotional processes in line with the functional properties of the gradient. Exploratory analyses suggested that ATD exerted a trend for an overall dispersion and variance of SMN-FPN hierarchical axis. Although these findings did not survive a strict multiple comparisons correction they tentatively complement the focal, corrected effects observed in the hippocampus-vmPFC-amygdala circuitry and may indicate that ATD modulates the intrinsic large-scale network architecture of the brain. Future studies in larger cohorts are needed to confirm the robustness of these global effects. At the large-scale network level, ATD changed gradient scores in subnetworks, consistent with findings on regional and network level. FPN critically involved in range of executive domains, including inhibitory control and emotion regulation, as well as the experience of affective arousal (Scolari et al. 2015; Zhuang et al. 2023; Liu et al. 2024). Alterations in FPN intrinsic architecture have been associated with the allocation of attentional resources towards threat-like information (Dainer-Best et al. 2017; Xin et al. 2021) potentially heightening susceptibility to negative emotion—a bias commonly observed in anxiety disorders (Bar-Haim et al. 2007; Nejati et al. 2021). The observations underscore the pivotal role of 5-HT, as a critical neural regulator which is strongly involved in network modulation and communication across the entire brain. On the other hand, this may underlie the diverse serotonergic functions from response to early pressure (Liu et al. 2021b) and stress (Lan et al. 2022) to cognitive regulation, external attention and motor control (Cools et al. 2008; Quist et al. 2003). Thus, our results provide a potential linkage between gradient architecture disruption and impairments in 5-HT system. However, we only observed the effect of ATD in the third gradient, possibly due to the relatively small sample size of participants, as compared to previous studies utilizing larger datasets (Sun et al. 2023; Xia et al. 2022b).

Findings of the present study need to be considered in the context of the following limitations. First, the current study was conducted exclusively with male participants, to reduce variance in the data related to sex differences in the 5-HT synthesis, which limits generalizability of the results. Future studies are required to extrapolate the findings to the broader population, including women, different age groups and clinical populations (anxiety or mood disorders). In addition, although our sample size is comparable to previous pharmaco-fMRI studies, it may still be modest for detecting genome-wide interaction effects, potentially limiting statistical power. These findings should therefore be considered exploratory and require replication in larger cohorts. Second, although we adopted the identical amino-acid mixture and dosing schedule evaluated in previous studies to lower plasma tryptophan levels, we did not collect peripheral blood samples to quantify these indices. Future work should therefore incorporate plasma tryptophan and TRP/LNAA assays to validate the neurochemical impact of the ATD challenge. Ultimately, elucidating the complete causal pathway from genotype to brain function will require multimodal approaches that integrate e.g. also molecular imaging maps as proposed e.g. in the REACT framework (Boucherie et al. 2023).

## Conclusion

In conclusion, the present study provides compelling evidence of the pivotal role of 5-HT in modulating intrinsic neural activity and connectivity within specific circuits closely related to anxiety regulation, with the individual variations in a TPH2 polymorphism altering susceptibility to the neural effects of transient serotonin depletion induced by ATD. This may account for the previously inconsistent effects of ATD, thereby identifying a potential genetic marker of vulnerability for anxiety-related conditions. Moreover, our study provides the first evidence of 5-HT’s impact on brain gradient architecture, underscoring its essential role in modulating network communication across the entire brain and offering a plausible explanation for the extensive functional repertoire of serotonin. Future research should incorporate more diverse samples and whole-brain analyses to corroborate and extend these findings, as well as explore additional genetic factors that may modulate serotonergic function.

## Supplementary Information

Below is the link to the electronic supplementary material.

Supplementary Material 1

## Data Availability

Data related to the study is available from the corresponding author upon reasonable request.
