# Supplementary material for: State and trait serotonin variations interact to shape the intrinsic connectivity and gradient architecture of the brain – a combined TPH2 genetics and tryptophan depletion study in men
Source: Psychopharmacology (Berl). 2026 Feb 24;243(11):2613–25. doi: 10.1007/s00213-026-07029-4 (PMC13710607; doi:10.1007/s00213-026-07029-4)
Supplement: Supplementary file 1 [file 213_2026_7029_MOESM1_ESM.docx]

Supplementary Information for

**State and trait serotonin variations interact to shape the intrinsic connectivity and gradient architecture of the brain – a combined TPH2 genetics and tryptophan depletion study in men**

**Authors**

Lan Wang^1,2#^, Congcong Liu^3#^, Xianyang Gan^1,2^, Keith Kendrick^1,2^, Weihua Zhao^1,2^, Christian Montag^4^, Ting Xu^5,6^*, Benjamin Becker^1,7^*

**Affiliations**

^1^The Center of Psychosomatic Medicine, Sichuan Provincial Center for Mental Health, Sichuan Provincial People's Hospital, University of Electronic Science and Technology of China, Chengdu, China

^2^School of Life Science and Technology, University of Electronic Science and Technology, Chengdu, China

^3^School of Psychology, Xinxiang Medical University, Xinxiang, China

^4^Department of Psychology, Ulm University, Ulm, Germany

^5^Faculty of Psychology, Southwest University, Chongqing, China

^6^Key Laboratory of Cognition and Personality, Ministry of Education, Chongqing, China

^7^Department of Psychology, The University of Hong Kong, Hong Kong, China

**^#^Shared first-authorship**

**^*^Shared correspondence**

Benjamin Becker

The University of Hong Kong,

Department of Psychology

E-mail: [ben_becker@gmx.de](mailto:ben_becker@gmx.de)

Ting Xu

Southwest University

Faculty of Psychology

E-mail: [tingxu.uestc@outlook.com](mailto:tingxu.uestc@outlook.com)

**Acknowledgments of funding and grants**

The work was partly supported by the STI 2030–Major Projects (Grant No.2022ZD0208500), National Natural Science Foundation of China (Grants No. 32250610208, 82271583) and a start-up funding from The University of Hong Kong. The funders had no further role in study design; in the collection, analysis and interpretation of data; in the writing of the report; and in the decision to submit the paper for publication.

**Declaration of conflicting interests**

The authors declared no potential conflicts of interest with respect to the research, authorship, and/or publication of this article.

**Supplementary Methods**

**Region of interest (ROI) definition**

The Neurosynth-based vmPFC mask that we used contained a total of 1117 voxels, detailed regions and voxels information see **Table S1** (list the brain regions with the number of voxel greater than 15).

**Table S1 Brain region and the number of voxels**

| Voxel | Automated Anatomical Labeling |
| --- | --- |
| 174 | Right frontal medial orbitofrontal gyrus |
| 152 | Left frontal medial orbitofrontal gyrus |
| 146 | Left rectus |
| 104 | Right rectus |
| 100 | Left superior frontal medial gyrus |
| 75 | Left pregenual anterior cingulate cortex |
| 44 | Right medial orbital frontal cortex |
| 35 | Left subgenual anterior cingulate cortex |
| 32 | Right superior frontal medial gyrus |
| 28 | Left olfactory cortex |
| 20 | Right pregenual anterior cingulate cortex |
| 16 | Right superior anterior cingulate corte |
| 15 | Right olfactory cortex |

**Supplementary Results**

To ensure that the reported findings were not confounded by head motion or clinical measures, all primary fALFF and FC analyses included the mean framewise displacement (FD) from both experimental sessions, alongside SAI, TAI, CTQ, and PSS scores, as nuisance covariates in the statistical model. The results remained consistent and robust after controlling confounds (See Table S2-S4).

**Table S2 Significant results for fALFF before and after controlling for covariates (p<0.001)**

| Condition | Peak coordinates (x, y, z) | Voxel size (k) | Anatomical label | T | p_FWE_ |
| --- | --- | --- | --- | --- | --- |
| Before | -33, -21, -18 | 6 | Left Hippocampus | 4.47 | 0.045 |
| After | -30, -18, -18 | 7 | Left Hippocampus | 4.58 | 0.044 |

Before: Before covariate control (mean FD, CTQ, PSS, STAI)

After: After covariate control (mean FD, CTQ, PSS, STAI)

**Table S3 Significant effects of ATD treatment on local functional connectivity (p<0.001)**

| Condition | Peak coordinates (x, y, z) | Voxel size (k) | Anatomical label | T | p_FWE_ |
| --- | --- | --- | --- | --- | --- |
| Before | 0, 30, -15 | 59 | Medial frontal gyrus | 4.10 | 0.021 |
| After | 0, 30, -15 | 63 | Medial frontal gyrus | 4.16 | 0.023 |

Before: Before covariate control (mean FD, CTQ, PSS, STAI)

After: After covariate control (mean FD, CTQ, PSS, STAI)

**Table S4 Significant interaction effects of treatment with genotype on local functional connectivity (p<0.001)**

| Cluster | Peak coordinates (x, y, z) | Voxel size (k) | Anatomical label | T | p_FWE_ |
| --- | --- | --- | --- | --- | --- |
| Before covariate control |  |  |  |  |  |
| Cluster 1 | 18, 0, -15 | 23 | Right amygdala | 4.19 | 0.017 |
| Cluster 2 | -24, 3, -18 | 48 | Left amygdala | 4.09 | 0.023 |
| After covariate control |  |  |  |  |  |
| Cluster 1 | -9, 0, -21 | 53 | Left parahippocampal gyrus | 4.49 | 0.010 |
| Cluster 2 | 18, 0, -15 | 15 | Right amygdala | 4.02 | 0.033 |
